# A national qualitative investigation of the impact of service change on doctors’ training during Covid-19

**DOI:** 10.1186/s12909-023-04143-1

**Published:** 2023-03-20

**Authors:** M. E. W. M. Silkens, K. Alexander, R. Viney, C. O’Keeffe, S. Taylor, L. M. Noble, A. Griffin

**Affiliations:** 1 Research Department of Medical Education (RDME), UCL Medical School, University College London, The Directorate, 74 Huntley Street, London, WC1E 6AU UK; 2 Centre for Healthcare Innovation Research, Department of Health Services Research and Management, City University of London, London, UK; 3 School of Medicine, University of Dundee, Dundee, UK; 4 UCL Medical School, University College London, London, UK; 5 Institute of Education, University College London, London, UK

**Keywords:** Postgraduate medical education, Medical training, Service reconfiguration, Learning environments, Case-study research

## Abstract

**Background:**

The Covid-19 crisis sparked service reconfigurations in healthcare systems worldwide. With postgraduate medical education sitting within these systems, service reconfigurations substantially impact trainees and their training environment. This study aims to provide an in-depth qualitative understanding of the impact of service reconfiguration on doctors’ training during the pandemic, identifying opportunities for the future as well as factors that pose risks to education and training and how these might be mitigated.

**Methods:**

Qualitative parallel multi-centre case studies examined three Trusts/Health Boards in two countries in the United Kingdom. Data were collected from online focus groups and interviews with trainees and supervisors using semi-structured interview guides (September to December 2020). A socio-cultural model of workplace learning, the expansive-restrictive continuum, informed data gathering, analysis of focus groups and coding.

**Results:**

Sixty-six doctors participated, representing 25 specialties/subspecialties. Thirty-four participants were male, 26 were supervisors, 17 were specialty trainees and 23 were foundation doctors. Four themes described the impact of pandemic-related service reconfigurations on training: (1) Development of skills and job design, (2) Supervision and assessments, (3) Teamwork and communication, and (4) Workload and wellbeing. Service changes were found to both facilitate and hinder education and training, varying across sites, specialties, and trainees’ grades. Trainees’ jobs were redesigned extensively, and many trainees were redeployed to specialties requiring extra workforce during the pandemic.

**Conclusions:**

The rapid and unplanned service reconfigurations during the pandemic caused unique challenges and opportunities to doctors’ training. This impaired trainees’ development in their specialty of interest, but also presented new opportunities such as cross-boundary working and networking.

**Supplementary Information:**

The online version contains supplementary material available at 10.1186/s12909-023-04143-1.

## Background

The Covid-19 pandemic in 2020 brought crisis for healthcare systems worldwide [1]. This sparked unplanned service reconfigurations, such as reducing footfall in care facilities by switching from in-person to virtual clinics [2, 3], a redistribution of staff to critical care [4], and delays in treatment [5]. Service reconfigurations in healthcare systems – defined as changes in those processes that affect what services are delivered and where [6] – are commonplace, and enable healthcare to keep up with technological, medical, and societal advancements and demands [7]. However, the Covid-19 pandemic triggered unprecedented reconfigurations affecting healthcare around the world [1]. As a result, there have been positive and negative long-term effects of the changes made. Virtual teaching, for example, improved the accessibility and feasibility of teaching and examinations in many settings [8]. At the same time, however, the pandemic caused stress, fatigue and anxiety which contributed to burnout amongst junior doctors [9]. In the United Kingdom (UK), in common with many other countries, postgraduate medical education (PGME), is workplace-based (i.e. trainees learn while they provide patient care). Hence these changes could introduce risks as well as opportunities to PGME, affecting quality of care and the mental wellbeing of future doctors [10].

Trainees, supervisors, policymakers and researchers have voiced concerns that the service reconfigurations implemented during the Covid-19 pandemic substantially impacted trainees and their training environment [2, 4, 10–14]. A dearth of patients limits trainees’ clinical and surgical exposure [12], virtual consultations limit trainees’ opportunities to benefit from teacher-trainee ‘bedside teaching’ [15] and trainees’ mental wellbeing may be adversely affected [13]. However, there may also be positive effects, such as opportunities for junior doctors to contribute to service reconfigurations and increased confidence when managing very unwell patients [4].

This study was positioned within a larger commissioned project researching the impact of service reconfiguration on PGME in the UK, which was initiated in 2019 [16]. The project investigated the impact of planned service reconfigurations (e.g. hospital mergers, regional reconfigurations and acute hospital reconfigurations) on doctors’ training [16]. As the onset of the pandemic led to sudden and profound unplanned service change, this provided the opportunity to gain an in-depth qualitative understanding of the following research questions: What are the effects of service reconfiguration during the pandemic on doctors’ training and what opportunities and risks does this pose for PGME? The objective of this study was to understand the implications of widescale and rapid service reconfiguration which may also reveal factors that can ensure that training can continue effectively during unplanned change.

## Methods

### Setting

This study was conducted in the UK, where all residents are entitled to healthcare free at the point of delivery provided by the National Health Service (NHS). NHS hospitals are run by NHS Trusts (England/Northern Ireland) or Health Boards (Scotland/Wales): these are organisational units that provide care in a geographical area via several hospital sites. This study focuses on PGME which occurs within the NHS.

### Context

In the UK, medical graduates must complete two years of foundation training, which continues their education of the basic knowledge, skills and competencies required to treat patients. After the foundation years, doctors continue as trainees under the supervision of clinical and educational supervisors for their specialist training in one of the sixteen main specialties or in general practice. PGME in the UK is undertaken within the NHS and must meet the training standards defined by the General Medical Council, the UK regulator of the medical profession.

During the Covid-19 pandemic the NHS and PGME were severely disrupted. During the period of data gathering (September-December 2020), there were government mandated measures to address rising Covid-19 infections, including working from home, limits on social gatherings, travel restrictions, and a lockdown in November 2020.

### Study design

This was a qualitative study researching multiple multi-centre case studies in parallel [17]. This approach was selected to explore many variables in the study settings in-depth, considering that the people and circumstances in this context are woven together and are not easily separated [17]. This helped to identify which underlying patterns, mechanisms and processes may have positively or negatively influenced training during service reconfiguration.

Thomas and Myers [18] noted that rigorous case study research defines a subject and an object. The subject refers to the case or cases used in the study. For the current study these were the three Trusts/Health Boards that were going through service reconfiguration because of the Covid-19 pandemic, as well as planned service changes (detailed in Study participants and data collection below). These Trusts/Health boards were situated in two countries of the United Kingdom and each Trust/Health Board governed a collection of NHS hospitals, of which a total of eight NHS hospital sites were covered in this study. The object refers to the analytical frame through which the subject was analysed [18]. For this study, the object was a socio-cultural model: the expansive-restrictive continuum.

### The expansive-restrictive continuum

The expansive-restrictive continuum was devised by Fuller and Unwin [19, 20] to account for modern day experiences of workplace learning for apprentices. It is a development of Lave and Wenger’s [21] situated learning theory which explores legitimate peripheral participation in communities of practice. The expansive-restrictive continuum was built on the notion that the opportunities and encouragement to learn may vary by workplace [19, 20]. It proposes a continuum along which pedagogical, organisational and cultural factors that are involved in apprenticeship learning are positioned, either on the ‘expansive’ end of the continuum or on the ‘restrictive’ side [19, 20]. Factors on the ‘expansive’ end are thought to benefit apprenticeships as they allow the learner to develop, whereas the factors placed on the ‘restrictive’ side inhibit apprenticeships, because they limit the development of the learner.

Fuller and Unwin developed several variations of the continuum [19]. For this study, we chose an existing version of the continuum that describes those dimensions of the organizational learning culture which play a role in shaping the quality of training environments (Table 1). The continuum was used to design the interview guides (Supplement 2) and subsequently applied during coding (described in Analysis below) to identify how the Covid-19-related service reconfigurations affected trainees’ training environments and what aspects of these environments were on the ‘expansive’ or ‘restrictive’ side of the continuum. This, in turn, provided insights for future opportunities as well as the factors that posed risks to PGME and how these might be mitigated. It was beyond the scope of this study to place training environment factors along the continuum, rather we identified at which end of the continuum these factors we positioned – either expansive or restrictive or both. This is in line with the most common use of the framework [20] and – considering the qualitative nature of this study – allowed us to focus on identifying what training environment factors were affected by the Covid-19 pandemic and how, rather than focussing on a quantification of where the factors would sit along the continuum. Throughout the study, the continuum was iteratively evaluated by the research team and where data brought up new themes not yet included in the continuum, these were added to guarantee the applicability of the model to the study setting.

**Table 1:** The expansive-restrictive continuum as applied to training environments, developed by Fuller and Unwin^a^

EXPANSIVE	RESTRICTIVE
New and varied skills encouraged	Limited and one-sided skill development
Expanded job design	Restricted job design
Rotations encouraged	Lack of workplace mobility
Supervisor as enabler	Supervisor as controller
Bottom-up approach to innovation	Top-down approach to innovation
Teamwork and collaboration valued	Rigid specialist roles
Communication encouraged	Communication stifled
Formative approach to evaluation	Summative approach to evaluation
Individual progression encouraged	Skill brought in externally

^a^Evans K, Hodkinson P, Rainbird H, Unwin L. Improving workplace learning: Routledge; 2007 (page 61)

### Study participants and data collection

For the larger commissioned research project from which this study derives, six purposefully selected and geographically spread case study sites were approached, and three sites agreed to take part [16]. In March 2020, the project was paused due to the rapid growth of Covid-19 infections in the UK. When the project was resumed in September 2020, the research team decided to consider the Covid-19 pandemic as a type of unplanned service reconfiguration and to investigate its impact on training as an addition to the original project. This study reports solely on this addition.

Data for this study were collected between September and December 2020. Data were collected from the same three case study sites participating in the larger project (details on the sites are presented in Supplement 1, Table S1). All case study sites went through significant service reconfigurations during the pandemic, such as: the expansion of intensive care facilities, introduction of new models of care, online or alternative consultations, delay or transfer of elective care, categorisation of hospital sites into ‘Covid-19 safe’ and ‘Covid-19 unsafe’ sites, increased working from home and shielding, introduction of personal protective equipment (PPE), changing rotas, and the introduction of wellbeing interventions.

For each case study we invited educational and clinical supervisors and doctors in training (foundation and specialty trainees) to ensure we included multiple perspectives, an approach widely valued in case study research as it ensures the study provides a holistic impression of reality [18]. Clinical and educational leads facilitated recruitment by forwarding study invitations and participant information sheets. Interested participants contacted the research team by email. One or more focus groups were organised for each participant group per case study. Participants who were unable to participate in these were offered an interview instead. Focus groups and interviews lasted approximately one hour and were conducted online via Microsoft TEAMS. Participants received a certificate of participation as an incentive.

A semi-structured interview guide was developed by the research team drawing on the themes of the expansive-restrictive continuum [19]. The guide was applicable to supervisors and trainees. It included two introductory questions and the following main topics: education during service reconfiguration, positive and negative impacts of service reconfiguration on training, available support, and quality assurance (Supplement 2). The guide was refined after the first two interviews through discussion within the research team. No major changes occurred, but some questions were re-ordered for better flow, or merged, and some questions were added as a prompt to guarantee that key aspects of the expansive-restrictive continuum (Table 1) were sufficiently covered.

### Ethics

The study was registered with UCL’s Data Protection Office on 28/08/19 (registration number Z6364106/2019/08/120) and approved by UCL Ethics Committee on 05/09/19 (project number 15745/003). All participants signed informed consent which was stored on UCL Data Safe Haven and participation was voluntary. All data was pseudo-anonymised.

### Analysis

Data were audio recorded using the recording function in Microsoft TEAMS or by using an app (Audacity), then transcribed and subsequently redacted to remove identifiers from the transcripts. QSR International’s NVivo 12 software was used to support the thematic qualitative analysis. A combination of inductive and deductive coding was used, meaning that the themes mentioned in the expansive-restrictive continuum (Table 1) were used to inform the deductive coding. Inductive coding was used to further refine the continuum, so that irrelevant themes were removed and meaningful themes were added to the framework. Aspects that were described as contributing positively to trainees’ learning, development and professional performance by participants were considered to be expansive, whereas aspects that were described as inhibiting trainees to learn, develop, and provide high quality care were considered to be restrictive. MS, CO, AG, KA, RV and LN interviewed, and the entire research team conducted the analysis. The coding was iteratively discussed and refined by the research team throughout data analysis (e.g. addition of newly emergent themes, deletion of redundant/not applicable themes, and rewording of existing themes to better fit the data). The adapted continuum is described in the results.

## Results

### Study participants

A total of 66 participants participated in the research: 42 from case study one, 14 from case study two and 10 from case study three. Twenty-six participants were supervisors, 17 were specialty trainees, and 23 were foundation doctors. Further details on the sample are presented in Table S2, Supplement 1.

### Expansive-restrictive effects on training

We identified four themes that described the expansive-restrictive impact of pandemic-related service reconfigurations on training: (1) Development of skills and job design, (2) Supervision and assessments, (3) Teamwork and communication, and (4) Workload and wellbeing. These themes and their particular significance of the case study sites are summarized below. Table 2 shows how the themes relate to the expansive-restrictive continuum (Table 1) and provides an overview of which themes from the original framework were found to be relevant for the current study (including examples), which themes were not mentioned by participants, and which themes were added based on data analysis. It is important to note that impacts could be expansive, restrictive, or both. Furthermore, effects on training were similar across case study sites, although this varied by specialty and grade of the trainee.

**Table 2:** The adapted expansive-restrictive continuum describing impacts on doctors’ training during the pandemic

EXPANSIVE	RESTRICTIVE	ORIGIN
**Development of skills and job design**		
New and varied skills encouraged - Online learning facilitated new knowledge and skills by increased flexibility and improved accessibility - Opportunities to take on leadership or take an active role in adapting learning and care provision during the pandemic facilitated new skills	Limited and one-sided skill development - Face-to-face learning moments and clinics were cancelled, limiting trainees’ learning and network opportunities	Original continuum
Expanded job design - Trainees were exposed to and learned about new ways of providing patient care, such as conducting effective phone consultations	Restricted job design - Workplaces with a lack of focus on training restricted trainees to routine care provision and prevented them from learning and working beyond their current post	Original continuum
Rotations encouraged - Cross-site working enabled trainees to observe in new settings, thereby picking up valuable knowledge	Lack of workplace mobility - When trainees were prevented from cross-site working due to the pandemic, they experienced a lack of exposure to new specialties or learning situations	Original continuum
**Supervision and assessments**		
Supervisor as enabler - More consultants were available on the wards during the pandemic, meaning that supervisors were better able to guarantee that trainees were getting the most out of their job - Supervisors engaged trainees in management and governance of patient care throughout the pandemic	Supervisor as controller - Shielding and consultant absences meant that trainees were unable to observe their supervisors and lacked teaching - Supervisors felt online teaching could be inconsistent and efficacy was impaired	Original continuum
Assessments adapted to the learning situation - Expansive examples were not mentioned in the current study	Rigid assessments - The pandemic reduced learning opportunities and made the planning and proper execution of assessments more difficult - When assessments were unchanged during the pandemic, trainees had difficulties fulfilling assessment requirements	Current study
**Teamwork and communication**		
Teamwork and collaboration valued - Trainees working in new but fixed teams were able to make new and meaningful connections	Rigid specialist roles - Trainees that were working in the very transient workforce during the pandemic were unable to build meaningful networks	Original continuum
Communication encouraged - Expansive examples were not mentioned in the current study	Communication stifled - Despite frequent emails, communication was unclear. This led to consultants and trainees being confused about the status quo for training and patient care	Original continuum
**Workload and wellbeing**		
Attention for (mental and physical) wellbeing - Increased feeling of camaraderie amongst trainees, leading to solidarity amongst the workforce of trainees	No attention for (mental and physical) wellbeing - Due to the high workloads, trainees were unable to take annual leave. Trainees were also exposed to risks whilst providing patient care (e.g. working without proper personal protective equipment). This reflected negatively on trainees’ mental wellbeing, causing stress, anxiety, and burnout	Current study

For the quotes presented in the results, the origin of the quotes is indicated using the case study number (Case1; Case2; Case3), the type of data collection (INT = interview; FG = focus group), and the type of respondent (S = supervisor; T = Trainee; FY = Foundation year trainee). For example, Case3_INT1T refers to a quote from case study 3, which was given during the first conducted interview with trainees at that site.

#### Theme 1: Development of skills and job design

All case studies had to cancel formal teaching (e.g. courses and lectures) and develop alternative ways of teaching such as e-learning. Participants mentioned many aspects of online learning that could be considered expansive, such as increased flexibility to join, improved access for the wider body of trainees, and a reduced need to travel. However, trainees were restricted in the amount of time available to attend due to high workloads during the pandemic, especially in busy specialties, and felt compelled to catch up in their own time. Furthermore, cancelled clinics impaired their learning, although trainees mentioned this was more prominent in some specialties (e.g. surgery) where workplace learning was more disrupted. Conversely, some other specialties were operating close to normal.

“Covid has had a negative impact in terms of reduced clinics. […] For the first wave of the pandemic, the registrars didn’t go to clinic for four months […] so that had a negative impact. And now with the second wave the clinics are up and running to a certain extent, but obviously telephone consultations are more prominent […] so Covid’s been very negative” (Case3_INT1T).

With regards to workplace-based learning, there were new ways of providing care, such as conducting phone consultations, which could be useful and enriching. Supervisors also said opportunities arose, allowing trainees to expand by learning new jobs and developing new skills.

“It’s actually been really useful to learn how to take a history and also get some form of examination from patients when you can’t see them […] and I think would actually be useful when we return to seeing patients in person as well, picking up on their verbal cues” (Case1_FG2F).

“They [trainees] learnt in a different way during that period and they learnt different things. Not just clinical things even but just management skills, leadership skills, working as a team, mentoring. All kinds of things that they did without even being told to do that was very impressive actually ” (Case1_FG4S).

Trainee jobs were redesigned throughout the pandemic and trainees mentioned an important aspect of this was that they were partly or full-time redeployed to those specialties experiencing the highest case load during the pandemic. Trainees highlighted the expansive elements of this, saying that cross-site working allowed them to see how things are done in various settings and to take elements of this learning back to their regular workplace. However, for other trainees such redeployments were restrictive as they had to leave their new specialty rotations behind to return to specialties they had already been exposed to previously, thereby impairing their skill development.

“So if I was a trainee back then I would have been redeployed at some days into [SPECIALTY] again, which I don’t think would be adding much because I’ve been already there, so yeah this would have impacted the training experience in a major way” (Case1_FG1T).

Foundation trainees were frequently redeployed to fill rota gaps and service needs, and this was said to be especially restricting as the focus on training was completely lacking. For most foundation trainees this meant they missed out on planned rotations, impairing their development of skills and networks in specialties they possibly planned to continue in their career. Similar challenges, however, were mentioned by trainees who were withheld from rotating during the pandemic, mainly to retain trainees in high pressure specialties. Similarly to those forced to redeploy to an unanticipated specialty, those who were prevented from rotating to an anticipated specialty were worried about missing out on exposure in their desired specialties. Supervisors, however, said they hoped the upcoming years would allow trainees to catch up and they also stressed that most redeployed trainees picked up new skills successfully and that the redeployments provided useful new learning.

“[SPECIALTY] trainees who have spent a lot more time in acute or general internal medicine at the moment rather than in their speciality, we do hope that over the coming years - as training goes on for more than just a year or two luckily – they will then rebound and get more time back in their speciality” (Case3_INT3T).

#### Theme 2: Supervision and assessments

Supervisors mentioned many restrictive aspects of supervising during the pandemic, such as maintaining consistency when supervising online and impaired efficacy due to a lack of experience of teaching online. They also said, however, that more consultants were available on the wards as care was centralised and staff was redeployed to high pressure specialties. They said that because of this, trainees must have felt that supervision was better than ever.

“Yeah on the ground being supervised by a consultant was definitively the best it’s probably ever been” (Case1_FG5S).

Trainees indeed were overwhelmingly positive about their supervision during the pandemic, saying their supervisors managed updates on guidelines and polices introduced throughout the pandemic and made sure trainees felt safe. As supervisors and trainees were working together more closely, supervisors mentioned they could act in trainees’ best interests more easily to ensure trainees got the most out of their job.

Trainees did describe some restrictive elements of supervision during the pandemic, saying that shielding supervisors meant they were unable to observe trainees or schedule face-to-face meetings.

“If some people are shielding, so for example one of my supervisors is shielding, I haven’t physically been able to see him, I’ve only met him once in 3 months, and that will probably be the case for another couple of months as well. And because they’re doing non face to face clinics they haven’t had a chance to actually observe me and see what I would be like” (Case2_FG1T).

To counteract these more restrictive elements on supervision, some supervisors reported introducing expansive, new opportunities for trainees such as engaging them in management and governance.

“So if there are any opportunities like management (inaudible) this is another way of doing it. So that is what I try to do, not only just clinical, they have to develop as a whole where they need to manage things, the governance. So there will be opportunities, we need to be flexible and we need to speak to the trainees I think more frequently and look and basically listen to their requirements, their needs” (Case1_FG5S).

Trainees were also very worried that the pandemic decreased the number of opportunities to adequately prepare for and complete assessments and caused further difficulties in gaining sign-off of competencies. This was especially the case in situations where the consultant workforce was more transient due to the pandemic, making it harder for trainees to have continuity in observations and discussing cases, thereby possibly failing to meet assessment requirements. Trainees whose assessments during the pandemic had been altered worried that they would lack important competencies as a direct result of completing these adjusted examinations instead of the original assessments.

“Again they are focussing on the major presentations, but let’s say if before they would have asked for 10 assessments for the acute adult presentations for example, right now they are asking for five, they are focussing on very specific presentations. So this might impact some trainees that they will not aim to have formal assessments for as much acute presentations as used to before the Covid, if it makes sense” (Case1_FG1T).

Although supervisors were hoping this would not have long term effects on their progression, trainees said they were more worried.

“I’ve become a bit more worried about my training and progression within my training, I’m more worried about exams and more worried about how I’ll progress in the next job” (Case1_INT2T).

#### Theme 3: Teamwork and communication

Due to the large shifts in the workforce, trainees said they had to familiarise themselves and collaborate with new teams during the pandemic. To some trainees this was expansive, leading to opportunities to get to know their supervisors better and to collaborate and network with new colleagues. This was especially true for those working in new but fixed teams during the pandemic.

Others mentioned the more restrictive elements, saying that establishing proper relationships and teamwork was harder in the more transient workforce. Trainees indicated that the size of organisations may be relevant here: it was easier to get to know a new team when the ward was smaller as opposed to a more isolating large unit. Covid-19 rules also impacted trainees’ ability to teamwork, with supervisors actively discouraging communal lunches and networking activities.

Teamwork was further complicated by inadequate communication about pandemic-related changes. This caused uncertainty amongst supervisors and trainees which, in turn, led to dissatisfaction. Although trainees indicated that frequent emails kept them updated on changes affecting clinical care and informed them when clinics resumed, they said the situation was still very confusing.

“It wasn’t very clear what exactly we should and should not be doing” […]. And you know it wasn’t communicated to us properly, and when we were not doing the appropriate things, we were being told off by other members of staff” (Case1_FG3T).

#### Theme 4: Workload and wellbeing

Pandemic-related configurations (e.g. shielding protocols) and increased patient volumes increased the already high workload in most specialties and as a result, trainees felt restricted in their ability to attend training opportunities. These high workloads and reduced opportunities negatively impacted trainees’ mental wellbeing. Trainees particularly struggled with the inability to take annual leave during the pandemic, increased uncertainty about training and progression, increased stress due to rising workloads, and anxiety about contracting Covid-19 or to bring the virus home to loved ones. Some trainees even felt as if their mental wellbeing was put in harm’s way during the pandemic as they were exposed to risks unnecessarily.

“I think we shouldn’t underestimate probably the emotional impact of Covid, the good and the bad […]. They [trainees] just don’t know where to go and they’re lonely, and it creates such anxiety, and then we feel them telling us they’re burnt out a lot of the time” (Case2_FG1S).

Trainees mentioned, however, that going through the pandemic together did increase the feeling of camaraderie. As everyone was going through the same situation, trainees considered this to be expansive, mentioning this was a bonding experience, which caused trainees to look out for each other and to feel solidarity.

## Discussion

### Main findings

This study investigated the impact of unplanned service reconfiguration on doctors’ training during the pandemic. Using the expansive-restrictive continuum relevant to medical training, we found impacts on trainees’ development of skills and job design, supervision and assessments, teamwork and communication, and workload and wellbeing. Impacts could be expansive, restrictive, or both, and these were similar across case study sites.

### Expansive opportunities and restrictive challenges of sudden change

Trainees experienced several expansive elements of service reconfigurations that resulted from the pandemic. For example, the move from a face-to-face model to an online model increased the flexibility of learning, allowing trainees to join from any location and, when lectures were pre-recorded, to learn at any time. Although the pandemic caused a sudden ‘push’ to move to online education, the concept of online education was not unheard of before the pandemic. Svoboda et al. [22] described how some medical schools and training programmes were slowly moving towards more self-directed learning activities pre-pandemic, using online methods such as flipped-classrooms [23] and videoed lectures. The benefits of this according to Svoboda et al. are more efficient personalised learning, allowing students to revisit learning events according to their personal needs [22]. However, participants acknowledged more restrictive elements too, such as the inability to network with their peers as well as supervisors in the new online set-up, something that has also been found in research [12]. To mitigate this, forms of interactive online learning groups that allow trainees to collaborate and build working relationships online may be beneficial for training programmes in the future [12].

Although effects varied by trainees’ specialty and grade, workplace-based learning was expanded with opportunities such as ways to learn about new patientcare pathways, leadership opportunities or quality improvement initiatives. These opportunities were impromptu due to the unplanned nature of service reconfigurations in the pandemic. Research confirmed the importance of trainees spending time on, for example, academic development and writing [12] or clinical qualifications [3]. Expansive elements of cross-site working were also mentioned, allowing trainees to observe best-practices and support the introduction of such practices in other settings. However, supervisors were noticeably more optimistic about the opportunities provided by cross-site working whereas for trainees the more restrictive side of clinic cancellations and frequent redeployments were more prominent, such as the lack of exposure to clinical cases and practices such as elective surgery. Such a lack of exposure may mean that the new generation of doctors is missing competencies and may require extended time to catch up on lost learning opportunities, thereby possibly affecting the workforce in the long-term [10]. This may also impair the recruitment of trainees to specialties.

Despite the increased availability of supervisors on the wards and expansive opportunities introduced by these supervisors (e.g. engaging trainees in management and governance of patient care), overall trainees felt supervisors were too busy or unable (e.g. because of shielding) to observe or sign-off trainees’ competencies. Trainees therefore felt they were restricted in their ability to pass required assessments and examinations. Furthermore, trainees felt that exams that were altered due to the pandemic might not prepare them adequately for their future career in medicine. Although supervisors felt more optimistic about the long-term impacts of altered examinations of trainees’ progression than trainees, ultimately these views raise significant concerns for trainees’ progression through training and implies that supervision and assessments were misaligned throughout the pandemic [24].

For some trainees, especially those working in fixed teams, their ability to network and build relationships was expansive, creating new meaningful connections with peers and supervisors. For most, however, the transient nature of their work in combination with online learning, meant their relationships were restricted. Remarkably, trainees hardly talked about the effects on the doctor-patient relationship. Bahadur et al. [11] described how a meaningful therapeutic relationship is established through connection between the doctor and the patient, something that was potentially inhibited during the pandemic due to the switch to online clinics.

Finally, trainees’ mental wellbeing was severely restricted by the pandemic. The pandemic caused a higher workload, causing trainees to miss out on opportunities and preventing them from taking annual leave. Despite a feeling of solidarity and camaraderie which was mentioned as expansive, the uncertainty about training progression, and anxiety about contracting Covid-19 or bringing it home to loved ones, caused significant distress in trainees. Similarly, literature reports on the provider distress, isolation, fear, and anxiety caused by the pandemic [25] and suggests the implementation of meeting sessions focusing on mindfulness and stress management skills [26] and virtual cafes [27].

### The expansive-restrictive continuum

The expansive-restrictive continuum proved a useful model to investigate the impact of unplanned, pandemic-related service reconfiguration on doctors’ training. Many of the concepts brought forward by the original framework, such as skills development, job design, workplace mobility, supervision, teamwork, and communication were applicable to doctors’ learning environment during the pandemic. The empirical work in this study has contributed theoretically to the expansive-restrictive continuum by identifying two new elements. The first is trainee assessment. We argue this is an important element to incorporate in the continuum when reviewing medical training, as assessments are a crucial part of trainees’ learning environment. In the context of pandemic-related service change, there was a clear mismatch between the drastic changes in the learning environment and the relatively unaltered assessments trainees were expected to undergo. The second element is trainee wellbeing. This represents the affective component of the learning environment [28], which refers to how trainees are treated in their learning environment and how this affects their mental health and wellbeing. A sense of camaraderie and feeling integral to a team were positive effects of the pandemic-related changes. Together with the cognitive (i.e. supervision and assessments) and instrumental (i.e. job design) components already represented by the expansive-restrictive continuum, the affective component has a powerful effect on trainees’ experience of the learning environment and is therefore crucial to be represented [28].

Moreover, we propose a modification of the model based on the results of this study. While the expansive-restrictive continuum positions elements in the learning environment either on the expansive or the restrictive side of the continuum, our results show that some elements (e.g. cancellation of clinics) can be both expansive (e.g. leading to other learning opportunities) and restrictive (e.g. leading to loss of competencies).

### Strengths and limitations

This study finds its strengths in the triangulation of data, investigating three case studies representing a broad geographical spread, and including both trainee (across all training grades and multiple specialties) and supervisor voices. The timing of the study, during the pandemic, meant that study participants shared their experiences in real-time. However, equally due to the pandemic, attendance at focus groups was unbalanced across sites. Although we offered individual interviews as an alternative to allow those who had to cancel last minute to participate later, the interactive nature of the focus groups was challenged by small groups as well as the online setting. This may have prevented more dynamic group discussion, impoverishing the quality of the data. However, we reached theoretical saturation towards the end of data collection, contributing to our confidence that our data represents a wide array of perspectives. Although this study is limited to the UK context, we expect results to be transferrable and useful for the wider medical education context, especially considering the many similarities with early reports on the effects of the pandemic on medical education worldwide [2, 4, 8, 10–13, 15, 25].

### Implications for research and practice

As the Covid-19 pandemic has impacted worldwide, it provides a unique opportunity to triangulate this research in other contexts. This is especially important as our results imply the relevance of context in determining whether service changes are expansive or restrictive. A longitudinal study may clarify ongoing effects and provide an understanding of how to best ensure that trainees keep developing and progressing, regardless of the changes their training is going through.

For those involved in doctors’ training during ‘pressure-cooker’ situations such as the pandemic, it is crucial to try to preserve those elements of training that safeguard trainees’ career progression and enable them to provide safe and high-quality patient care. This can be achieved my emphasizing expansive elements of training and investing time and resources in reducing or resolving the impact of the more restrictive elements of training. Prioritising education, training, and supervision even in high-pressure environments is crucial to guarantee that trainees are exposed to a variety of learning opportunities so they can develop a breadth of knowledge and skills. Supporting trainees in building meaningful networks can further help this process along. Creating a sense of solidarity, paying attention to the physical and mental wellbeing of doctors including appropriate mental health screening and offering mental health resources and support [29], and keeping trainees (and the wider workforce) informed about changes and their envisioned effects on training and patient care can support trainees through the challenging times of a wholescale, rapid service reconfiguration.

## Conclusions

This study shows that service reconfigurations during the Covid-19 pandemic facilitated as well as hindered doctors’ development of skills and job design, supervision and assessments, teamwork and communication, and workload and wellbeing. These impacts varied across sites, specialties, and trainees’ grades. The unplanned service reconfigurations caused unique challenges, such as redeployment to specialties requiring extra workforce during the pandemic which impaired trainees ability to develop in their specialty of interest, but also provided opportunities to doctors’ training, such as cross-boundary working and networking.

## Supplementary Information

**Additional file 1: Supplementary material.** A table presenting case-study characteristics and the interview schedule used in the study.

## Data Availability

The datasets generated and analysed during the current study are not publicly available due to a risk of compromising individual privacy. As per guidelines from our ethics committee and data protection officer, our participants signed consent to agree their data will not be shared outside of the research team. Our research materials are available in the supplementary material and more detailed questions regarding the data used can be directed towards the corresponding author.

## Abbreviations

NHS: National Health Service
PGME: Postgraduate Medical Education
UK: United Kingdom
