# Supplementary material for: A national qualitative investigation of the impact of service change on doctors’ training during Covid-19
Source: BMC Med Educ. 2023 Mar 20;23:174. doi: 10.1186/s12909-023-04143-1 (PMC10027255; doi:10.1186/s12909-023-04143-1)
Supplement: Supplementary file 1 — Additional file 1: Supplementary material. A table presenting case-study characteristics and the interview schedule used in the study. [file 12909_2023_4143_MOESM1_ESM.docx]

**Supplementary material**

**Supplement 1: Tables**

**Table S1.** Case-study characteristics.

|  | Country | Number of hospital sites | Number of sites with trainees | Number of trainees |
| --- | --- | --- | --- | --- |
| Case-study 1 | 1 | 3 | 3 | Two sites between 100-199; one site <100 |
| Case-study 2 | 1 | 3 | 2 | One site >200; one site <100 |
| Case-study 3 | 2 | 3 | 3 | Two sites >200; one site between 100-199 |

**Table S2.** Study participant characteristics.

| **Trust/Health Board ID (total N participants)** | **Type of participant (N)** | **Focus group/interview (identifier)** | **Gender** |  | **Specialty** |
| --- | --- | --- | --- | --- | --- |
|  |  |  | Male (N) | Female (N) |  |
| 1 (42) | Supervisors* (17) | Case1_FG1S | 1 | 1 | Nephrology, Paediatrics |
|  |  | Case1_FG2S | 5 | - | Anaesthetics, emergency medicine, A&E (2x), medicine |
|  |  | Case1_FG3S | 1 | 1 | Older people medicine, nephrology |
|  |  | Case1_INT1S | 1 | - | Vascular surgery |
|  |  | Case1_FG4S | 1 | 3 | Respiratory medicine, medicine, neurology, acute medicine |
|  |  | Case1_FG5S | 3 | - | Paediatrics (2x), obstetrics & gynaecology |
|  | Core and specialty trainees (9) | Case1_FG1T | 1 | 1 | Internal medicine, emergency medicine |
|  |  | Case1_INT1T | 1 | - | Internal medicine |
|  |  | Case1_INT2T | - | 1 | Anaesthetics |
|  |  | Case1_FG2T | 2 | 1 | Paediatrics, medicine, GP |
|  |  | Case1_FG3T | 2 | - | Anaesthetics, endocrinology |
|  | Foundation trainees (16) | Case1_FG1F | 5 | 7 | Medicine (4x), surgery (4x), psychiatry (2x), geriatrics, orthopaedics |
|  |  | Case1_INT1F | 1 | - | Acute medicine |
|  |  | Case1_FG2F | 1 | 2 | Surgery, GP, medicine |
| 2 (14) | Supervisors** (4) | Case2_INT1S | - | 1 | Medical education |
|  |  | Case2_FG1S | - | 3 | Obstetrics & gynaecology, A&E, Medical education |
|  | Core and specialty trainees (3) | Case2_FG1T | - | 2 | Obstetrics & gynaecology, medicine |
|  |  | Case2_INT1T | 1 | - | GP |
|  | Foundation trainees (7) | Case2_INT1F | 1 | - | Geriatrics |
|  |  | Case2_FG1F | 2 | 4 | GP, paediatrics, geriatrics, medicine (2x), A&E |
| 3 (10) | Supervisors** (5) | Case3_FG1S | 3 | - | Plastic surgery, biochemistry, orthopaedics |
|  |  | Case3_INT1S | 1 | - | Diabetes and endocrinology |
|  |  | Case3_INT2S | - | 1 | Physician |
|  | Core and specialty trainees (5) | Case3_INT1T | - | 1 | Medicine |
|  |  | Case3_INT2T | - | 1 | Geriatrics |
|  |  | Case3_FG1T | 1 | 1 | Infectious diseases (2x) |
|  |  | Case3_INT3T | - | 1 | Gastroenterology |

* Supervisors (includes Directors & Associate Directors of Medical Education, Education and Clinical Supervisors).

**This focus group contained one specialty trainee (medicine).

**Supplement 2: Interview schedule for supervisors/trainees**

**Introductory questions:**

1. Could you briefly tell me about your experience of working at this Trust/Health Board which is going through service reconfiguration?
2. What is the biggest challenge/s during service reconfiguration?

- Were you able to overcome them? How?
- What are your priorities during this time?

**Main questions:**

1. Do you think that postgraduate medical training is adequately considered at times of service reconfiguration?

- Prompt: Why do you say that? [Other priorities e.g. targets]
- Prompt: In your opinion, are there any factors that you think are important that are not generally considered?

1. How could medical education and training be put on the wider agenda during times of service reconfiguration?
2. Do you think service reconfiguration has impacted on your experience of [training] [supervising]?

- Prompts:
  - Learning/support of learning
    - Are there adequate opportunities to learn? [covering the curriculum (formal and informal), reflection, study leave].
    - Are there adequate opportunities to develop new skills?
    - Is there active support for [doctors in training] [supervisors]? Who provides this? (Trust leadership, Departmental leads, Supervisors)?
    - Are you/doctors in training encouraged to develop holistically?
  - Relationships
    - Are you/doctors in training encouraged to network with colleagues in other departments?
    - Do you feel your role as [doctor in training] [supervisor] is valued?
    - Are you/doctors in training encouraged to work in teams or is the work more isolating?
    - Has service reconfiguration impacted on relationships at work?
  - Workload
    - Is the workload manageable? (amount/organisation of work/rota gaps)
    - Do you think that service reconfiguration has impacted on supervision time – how? In hours and out of hours?
  - Communication/Culture
- Have you seen service reconfiguration impact on wellbeing or morale?
- Has there been good communication with you about service reconfiguration?
- If you have made comments about this do you feel these have been heard?
- Has the service reconfiguration been well led? Why?
- Is there a hierarchical approach/top-down approach to managing change?

1. Could you give any examples of how service reconfiguration has improved your experience? Has service reconfiguration had negative impacts on you as a [doctors in training] [supervisor]?

- Prompt: What were the reasons for this?
- Prompt: What affect did it have?

1. Are there other factors/pressures outside service reconfiguration that might influence the changes you see occurring in your Trust?
2. Do you have any insights about how risky training environments could be identified?
3. What support do you think is important for you in your role as [doctor in training] [supervisor] when service reconfiguration is occurring?
   - Prompt: Why do you think so – why more so than when service is stable?
4. And what support do you think would be helpful to put in place to ensure high quality learning environments at times of change?
